# Combustion, Respiration and Intermittent Exercise: A Theoretical Perspective on Oxygen Uptake and Energy Expenditure

**DOI:** 10.3390/biology3020255

**Published:** 2014-03-28

**Authors:** Christopher B. Scott

**Affiliations:** Exercise, Health and Sports Sciences Department, University of Southern Maine, Gorham, ME 04038, USA; E-Mail: cscott@usm.maine.edu

**Keywords:** oxygen uptake, anaerobic energy costs, glycolysis, EPOC

## Abstract

While no doubt thought about for thousands of years, it was Antoine Lavoisier in the late 18th century who is largely credited with the first “modern” investigations of biological energy exchanges. From Lavoisier’s work with combustion and respiration a scientific trend emerges that extends to the present day: the world gains a credible working hypothesis but validity goes missing, often for some time, until later confirmed using proper measures. This theme is applied to glucose/glycogen metabolism where energy exchanges are depicted as *conversion* from one form to another and, *transfer* from one place to another made by both the anaerobic and aerobic biochemical pathways within working skeletal muscle, and the hypothetical quantification of these components as part of an oxygen (O_2_) uptake measurement. The anaerobic and aerobic energy exchange components of metabolism are represented by two different interpretations of O_2_ uptake: one that contains a glycolytic component (1 L O_2_ = 21.1 kJ) and one that does not (1 L O_2_ = 19.6 kJ). When energy exchange transfer and oxygen-related expenditures are applied separately to exercise and recovery periods, an increased energy cost for intermittent as compared to continuous exercise is hypothesized to be a direct result.

## 1. Introduction

Antoine Lavoisier (1743–1794) focused on combustion and respiration as processes that first and foremost took something from the air as opposed to providing something to the air (i.e., phlogiston) as many of his time had asserted [1]. His measurements concerning combustion pre-date those of respiration and appear to have laid the ground work for his prescient interpretation of biological respiration: respiration was, he declared, a slowed down version of combustion. The world gained a valuable hypothesis. However, what exactly was our so-called father of chemistry measuring during these experiments? With considerable use of hindsight, it appears that *valid* quantitative measurements of oxygen (O_2_) likely went missing for some time.

## 2. Combustion

Lavoisier approached combustion by carefully weighing reactants and products. Instead of losing weight the ash of burned phosphorus actually increased. Lavoisier postulated a combining of phosphorous with “something” taken from the air [2], reasoning that Joseph Priestley’s acid producer—oxygen (O_2_)—was that something [1]. An added presence following combustion can certainly be indicated with a state-of-the-art 18th century balance-scale, yet quantifying exactly how much O_2_ certainly went missing because atomic and molecular weights were unknown at the time.

A case can be made that Lavoisier’s measurements of respiratory gas exchanges using animal and human subjects primarily involved carbon dioxide (CO_2_). After reading contemporary analyses of Lavoisier’s respiration experiments, it is difficult to recognize whether or not the measurement of O_2_ took place [1,2,3,4,5,6]. Even so, with the results of his combustion experiments Lavoisier had and the world gained a credible working hypothesis: both combustion and respiration took oxygen from the air. It is of interest that Atwater’s and Rosa’s heat calorimeter, that set the precedent for the validity of O_2_ uptake in the estimation of human energy expenditure, became operational in 1897 but did not *directly quantify* O_2_ uptake until subsequent modification in 1903–1904 [7]. 

Moreover, “… [Lavoisier] discovered that carbon dioxide is formed by a union of carbon and oxygen; and noting the consumption of oxygen and production of carbon dioxide in respiration, he advanced, for the first time, the view that the one was concerned in the production of the other” [5] (p. 135). Again, the world gained another clever working hypothesis: O_2_ uptake and CO_2_ production were directly related, the disappearance of one (O_2_) correlated to the presence of the other (CO_2_) in a perfect 1:1 relationship. Lavoisier’s hypothesis concerning O_2_ uptake and its relationship to CO_2_ production again fulfills the “something gained” theme presented here. The “something missing” theme also emerges, the energy cost and gas exchange differences between glucose and fat oxidation. The Respiratory Exchange Ratio (RER; CO_2_ ÷ O_2_) recognized a full century later, depicted a perfect one-to-one relationship with O_2_ uptake and CO_2_ production only when glucose was oxidized (Table 1). Not so for pure fat oxidation, or some mixture of both (the latter being the more realistic when appropriated to most diets).

**Table 1:** Glucose and fat oxidation.

**Glucose oxidation: **C_6_H_12_O_6_ + 6O_2_ → 6H_2_O + 6CO_2_ + ~2802 kJ RER = 1.00
**Fat oxidation:** C_16_H_32_O_2_ + 23O_2_ → 16H_2_O + 16CO_2_ + ~10,040 kJ RER = 0.70

Based on the measured volume of O_2_ consumed, less heat is lost with fat oxidation (1 L O_2_ = 19.6 kJ) and more with glucose oxidation (1 L O_2_ = 21.1 kJ). Why does glucose appear to have greater energy availability per liter of O_2_? Thermodynamic laws literally sprung up in the middle of the 19th century, providing a vivid understanding of the inherent in-efficiency of energy exchange. Based on combustion experiments emphasis has been placed on energy conversion—from one form to another—as the chemical bonds of reactants are converted to those of products [8]. Energy exchanges however, can invoke two different scenarios: from one form to another—*conversion* and, from one place to another—*transfer*. Neither is perfect and in-perfectiveness costs, energy exchanges are inefficient. The heat loss difference between glucose and fat is approximately 6%–7% using contemporary liters of O_2_ and CO_2_ measurements. Combustion scientists interpret such a difference as being somewhat inconsequential [9,10,11]; biologists and physiologists should think otherwise.

Combustion is defined as the single (and violent) act of oxygen’s combination with carbon and hydrogen, typically measured in the confines of a bomb calorimeter; energy conversion is but energy transfer is not part of the exchange. To the contrary product and reactant transfer from enzyme to enzyme takes place along a biochemical metabolic pathway. The inherent in-efficiency of energy exchange devices/mechanisms/machines—knowledge gained by engineers—has been only slowly appropriated to the study of biology [12,13]. In fact the concept of metabolic biochemical pathways went missing until around 1940, after glycolysis had been elucidated in full [14] (p. 349). 

## 3. Respiration

Metabolism is defined and represented by open system not closed system thermodynamics. Glucose related metabolic ATP re-synthesis involves several enzymes where reactants and products undergo *conversion and transfer* from enzyme-to-enzyme (*i.e.*, compartment-to-compartment) in what must be a highly ordered fashion [15,16,17,18,19]. Random diffusion is not solely at play here, and the creation and maintenance of order between cellular compartments costs. It may, thus, be hypothesized that metabolic energy exchanges are the result not only of chemical conversion—reactant breakdown with the formation of product—as every published text of Chemistry is aware, but also by the in-efficiency of an operational metabolic pathway that attempts to organize reactant-to-product transfer [18,19,20] (in eukaryotic plant cells intracellular flow—cyclosis—has been demonstrated). If individual enzymatic reactions and metabolons are each considered to be independent energy exchange “devices”, placed in-series, then they have the potential to influence a metabolic pathways overall efficiency (a metabolon is defined here as a single complex of several sequential enzymatic steps) (Table 2 and Table 3).

**Table 2:** Each numeral represents an enzymatic reaction (compartment) at 95% efficiency.

1 = 95% (efficiency of exchange)
1 × 2 = 90%
1 × 2 × 3 × 4 × 5 = 77%
1 × 2 × 3 × 4 × 5 × 6 × 7 × 8 × 9 × 10 = 60%

**Table 3:** Two metabolons are shown, each consisting of five enzymes; each metabolon (compartment) is 95% efficient.

[1 − 2 − 3 − 4 − 5] × [6 − 7 − 8 − 9 − 10] = 90% (efficiency of exchange)

True or not, the hypothesis that metabolic efficiency can be portrayed in part via in-series engineering can be presented, but direct data to confirm such a hypothesis goes missing. Indirectly, perhaps not. The concept of metabolic in-efficiency is interpreted later in the context of an energy cost difference between intermittent and continuous exercise.

Glucose degradation, as the field of biochemistry subsequently discovered, contains the metabolic pathways (compartments) of anaerobic glycolysis and aerobic (mitochondrial) respiration, both of which serve to re-synthesize ATP. Fat oxidation contains only an aerobic respiratory component. The metabolic differences between substrates should be recognized in terms of how overall energy costs are quantified. Josiah Willard Gibbs (1839–1903) contribution to science and chemistry in particular, was the concept of “available” energy (G). In addition, what a gain it was: energy *is* conserved. One would be hard-pressed to find any biochemistry textbook void of a listing of the Gibbs energy changes, reaction by reaction, along any metabolic pathway.

Gibbs breakthrough was based on the use of entropy (*S*), a concept that best describes how energy is dissipated or dispersed throughout a system [21,22]. Strangely enough (or perhaps not) contemporary biochemical texts rarely provide enthalpy (∆H) and entropy (∆*S*) data. That information, in regard to both the overall metabolic pathways and their individual reactions, often goes missing. Gibbs proof of the 1st thermodynamic law indicates that changes in Gibbs energy (∆G) are “driven” with changes in enthalpy (∆H) *and* entropy (∆*S*). 

**Figure 1:**
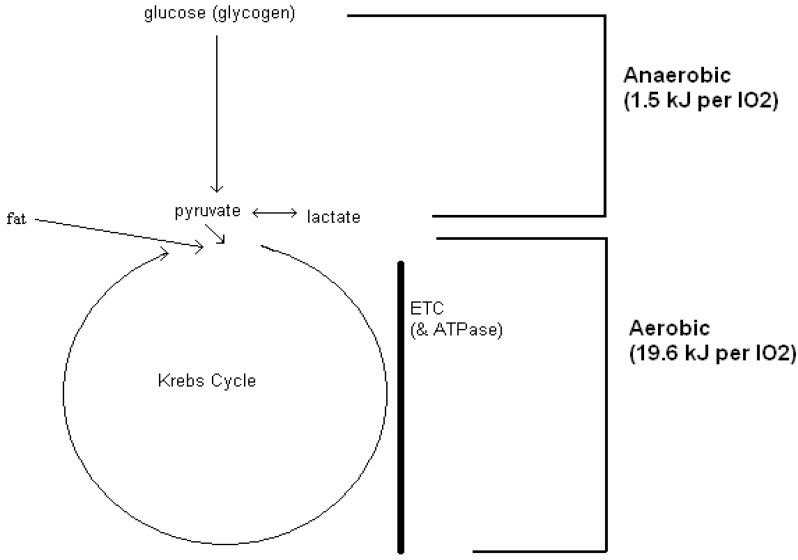
The complete metabolic degradation (biological oxidation) of glucose is converted to an estimate of energy expenditure as 21.1 kJ per liter of measured O_2_ uptake. In this example, the origins of that energy expenditure are hypothesized to have an anaerobic (glycolytic) component at 1.5 kJ per liter of O_2_ uptake and, an aerobic component at 19.6 kJ per liter of O_2_ uptake. Via aerobic respiratory-only metabolism, fat and lactate oxidation have no anaerobic component at 19.6 kJ per liter of O_2_ uptake. ETC = electron transport chain. ATPase = mitochondrial ATPase.

In describing glycolysis Minakami and de Verdier state, “It might be considered that of the two terms of Gibbs free energy expressed as *∆G = ∆H − T ∆S*, glycolysis is driven mainly by the entropy term (*∆S*) until the last step and the reaction is finally driven by the enthalpy term (∆*H*)…” [23] (p. 460). What is gained here is the knowledge of the overall enthalpy change associated with anaerobic glycolysis, from glucose-to-lactate. What goes missing is an energy cost measure (or estimate) of glucose-to-pyruvate degradation that precedes mitochondrial pyruvate oxidation. In the absence of lactate formation, the energy expenditure of glycolytic metabolism that immediately precedes mitochondrial metabolism is better quantified by entropy, and neither direct calorimetry (enthalpy) nor a measure of O_2_ uptake (indirect calorimetry) is able to quantify those entropy changes that characterize glucose-to-pyruvate degradation. However, a working hypothesis is gained when anaerobic (glycolytic) energy exchanges are acknowledged as an “extra” part of a respiratory O_2_ uptake measurement (Figure 1) [24]. 

## 4. Intermittent and Continuous Exercise

**Figure 2:**
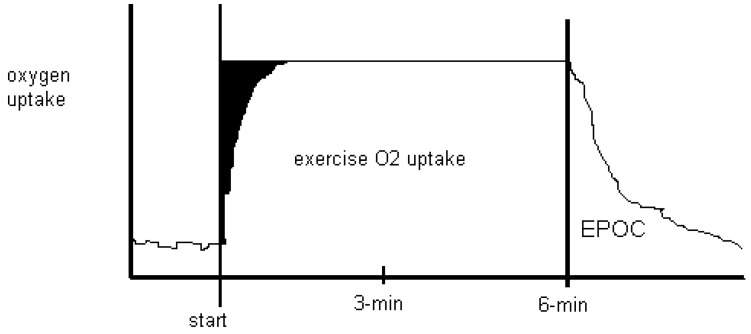
Oxygen uptake for six-minutes of low-intensity continuous steady state exercise with recovery is depicted. Anaerobic energy costs are shown in black as an *oxygen deficit* at the start of exercise, as it takes time to achieve a steady state O_2_ uptake. After exercise, recovery takes place where O_2_ uptake returns to resting levels. Traditionally called the O_2_ debt, the name was changed to excess post-exercise oxygen consumption (EPOC) in a qualitative attempt to dismiss lactate as being causal to recovery O_2_ uptake [25].

Exercise energy expenditure can include a measure of the O_2_ consumed in the recovery from that exercise. Utilization of the limited ATP and creatine phosphate (CP) stores within working skeletal muscle is considered part of anaerobic energy costs and the restoration of these two high-energy phosphates as part of aerobic mitochondrial respiration during recovery was indicative of a repayment (O_2_ debt) hypothesis. Based on this information exercise scientists wanting to estimate anaerobic energy costs have a choice, measure the size of the O_2_ deficit at the “front end” of exercise or, start from the “tail end” of exercise as part of an O_2_ debt (Figure 2). ATP, CP stores and utilization (and O_2_ stores too) cannot be twice accounted for by quantifying both the O_2_ deficit and debt.

However, what about anaerobic glycolytic energy expenditure that accompanies ATP re-synthesis during exercise? In fact, most pyruvate and much (but certainly not all) of the lactate produced within working skeletal muscle are oxidized as fuel. When the energy expenditure of lactate oxidation is interpreted as 21.1 kJ per liter of O_2_ consumed, the anaerobic glycolytic component can be accounted for twice, being part of the estimate of both exercise and recovery O_2_ uptake (Figure 1). In order to eliminate cause and effect concerning lactate production and the O_2_ debt respectively, exercise science gained a *qualitative* term that replaced “O_2_ debt”: excess post-exercise oxygen consumption or EPOC [25]. What continues to go missing is any change to the actual *quantification* of energy expenditure, whatever name the measurement of recovery O_2_ uptake goes by.

The 1.5 kJ difference per liter of O_2_ consumed between fat and glucose oxidation (Figure 1) identifies the quantitative presence of anaerobic glycolysis, whether it is entropy driven or enthalpy driven [20,24]. Removing the anaerobic glycolytic component from EPOC—where 1 L O_2_ = 19.6 kJ − eliminates the re-accounting of glycolysis when recovery energy expenditure is estimated by a measure of O_2_ uptake and fueled by fat or lactate oxidation. What continues to go missing are direct measurements to verify this within working and recovering skeletal muscle. Substrate disappearance is unfortunately best identified under steady state not non-steady state intermittent conditions. Again, indirect evidence supports a quantitative O_2_ uptake difference between intense intermittent and lower intensity continuous exercise.

**Figure 3:**
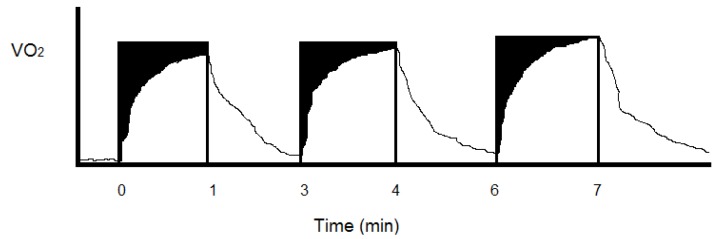
Divide continuous steady state aerobic exercise (Figure 2) into work equivalent or paired higher intensity intermittent periods and the total energy costs tend to increase. This energy cost increase is hypothesized to result from potential anaerobic (black) and aerobic (white) in-series metabolic inefficiency at the onset of each exercise bout and the increased O_2_ uptake of multiple recovery periods when lactate and fat (at 1 L O_2_ = 19.6 kJ) as opposed to glucose (at 1 L O_2_ = 21.1 kJ) are oxidized to meet energy demands. With intermittent resistance exercise, the summed recovery O_2_ uptake after each lifting period can greatly exceed the oxygen consumed during the actual weight lifting periods [30].

Divide a continuous single bout of steady state exercise into intermittent bouts of equivalent intensity and overall work [26,27] or higher intensity intermittent bouts with equivalent overall work [28] and the oxygen cost of the intermittent bouts can increase (Figure 3). Factor in anaerobic energy costs and a larger difference is found [29]. Based on this information steady state exercise does not provide a valid model of intermittent exercise costs. Why does intermittent exercise cost more than continuous exercise? 

As it is well known that the extent of recovery O_2_ uptake results from a possible multitude of physiological mechanisms that come at a cost [25], one explanation would be that multiple recovery periods in-and-of themselves would increase overall energy demands. The energy cost hypotheses presented here offer two additional explanations: (1) In-series inefficiency (Table 2 and Table 3) may result from the saltatory recruitment of both aerobic and anaerobic (glycolytic) metabolic pathways during multiple intermittent exercise bouts. With continuous exercise these pathways are recruited in tandem only at the onset of a single period of exercise. In fact, glycogen-lactate anaerobic energy cost contributions do indeed diminish with repeated exercise bouts, perhaps in an attempt to improve overall efficiency (or conserve glycogen stores) [26,30] and (2) Glycolysis appears to play little to no role in recovery. With lactate and fat oxidation fueling recovery, containing lower energy per liter of oxygen (as compared to glucose) (Figure 1), a greater amount of oxygen is subsequently consumed to meet energy demands during the multiple recovery periods that trail intermittent exercise bouts (Figure 3). 

As muscle glycogen stores are the preferred fuel of higher intensity exercise and fat oxidation is all-aerobic, exercise specialists have long promoted lower intensity continuous aerobic-type exercise as the best program for losing body fat. However, scientific and anecdotal research indicates the opposite—high intensity intermittent exercise invokes greater body fat losses as compared to continuous exercise [31,32]. When exercise and recovery are represented by separate O_2_ uptake interpretations, the energy exchange differences between intermittent and continuous exercise help explain this irony. Resistance training studies for example, reveal that the exercise and recovery O_2_ uptake profiles are in fact opposite those of continuous exercise [30]: as compared to continuous exercise, recovery after intermittent resistance exercise bouts can be summed to represent by far the largest O_2_ uptake component [33]; with continuous steady state work, exercise O_2_ uptake always exceeds recovery O_2_ uptake. A direct corollary is the design of intermittent exercise programs for body fat loss where emphasis is placed both on the recovery periods as well as the actual exercise.

## 5. Conclusions

As energy exchange devices, the metabolic pathways invoke both energy conversion (from one form to another) and energy transfer (from one compartment to another). Moreover, the anaerobic and aerobic energy exchange components of glucose metabolism can be represented by two different interpretations of an O_2_ uptake measurement: one that contains a glycolytic component (1 L O_2_ = 21.1 kJ) and, one that does not (1 L O_2_ = 19.6 kJ). These hypotheses help model the increased energy costs of intermittent as compared to continuous exercise. 
